# Post-traumatic amnesia is associated with increased arousal and reactivity symptoms following acquired brain injury

**DOI:** 10.3389/fcogn.2026.1880462

**Published:** 2026-07-28

**Authors:** Alessia Quinzi, Valentina Bandiera, Gaia Galluzzi, Alessandro von Gal, Maria De Luca, Nicoletta Fallarino, Paola Ciurli, Dolores Villalobos, Benedetta Ferrazza, Virginia Sordi, Elisa Berardi, Rita Formisano, Cecilia Guariglia, Umberto Bivona

**Affiliations:** 1 Azienda USL-IRCCS, Reggio Emilia, Italy; 2 Department of Human Science, LUMSA University, Rome, Italy; 3 Department of Psychology, PhD Program in Behavioral Neuroscience, “Sapienza” University of Rome, Rome, Italy; 4 IRCCS Fondazione Santa Lucia, Rome, Italy; 5 Department of Experimental Psychology, School of Psychology, Complutense University of Madrid, Madrid, Spain; 6 Center for Cognitive and Computational Neuroscience, Complutense University of Madrid, Madrid, Spain; 7 Institute of Knowledge Technology, Complutense University of Madrid, Madrid, Spain; 8 Department of Medicine and Health Sciences “Vincenzo Tiberio”, University of Molise, Campobasso, Italy

**Keywords:** acquired brain injury, anosognosia, functional disability, impairment in self-awareness, post-lesional amnesia, post-traumatic amnesia, post-traumatic stress disorder, post-traumatic stress symptoms

## Abstract

**Background:**

Post-traumatic amnesia is a common consequence of acquired brain injury and is characterized by impaired autobiographical memory for the traumatic event. For this reason, post-traumatic amnesia has traditionally been considered to reduce the risk of post-traumatic stress disorder, particularly symptoms based on explicit recollection of the trauma. However, post-traumatic stress symptoms may also emerge in the absence of conscious memory of the event.

**Objectives:**

To examine whether post-traumatic amnesia is associated with different patterns of post-traumatic stress symptoms following acquired brain injury.

**Methods:**

Fifty-six patients with acquired brain injury were divided into two groups: patients with post-traumatic amnesia (n = 31) and patients without post-traumatic amnesia (n = 25). Post-traumatic stress symptoms were assessed using the Structured Clinical Interview for Diagnostic and Statistical Manual of Mental Disorders – Clinician Version. Functional disability and self-awareness were also evaluated.

**Results:**

Post-traumatic stress disorder diagnoses were rare in both groups. However, patients with post-traumatic amnesia showed significantly higher arousal/reactivity symptoms than patients without post-traumatic amnesia, whereas no significant group differences emerged with regard to the dimension of intrusion, avoidance, negative alterations in cognition and mood, or general anxiety levels. Time since injury was positively associated with arousal symptoms in both groups. Functional disability predicted arousal only in patients without post-traumatic amnesia, while impaired self-awareness was negatively associated with arousal in the overall sample.

**Conclusion:**

The present findings challenge the traditional “amnesia-protection” hypothesis and support a more nuanced understanding of post-traumatic stress symptoms following acquired brain injury. Trauma-related emotional responses may persist despite impaired autobiographical memory for the traumatic event, highlighting the need for multidimensional approaches to the assessment of post-traumatic stress symptoms in this population.

## 1 Introduction

Acquired brain injury (ABI) is an umbrella term encompassing both traumatic brain injury (TBI), resulting from external mechanical forces (e.g., motor vehicle accidents, falls, or violence), and non-traumatic brain injury caused by internal pathological processes such as stroke, anoxia, infections, or neoplasms (Goldman et al., 2022). These conditions disrupt normal brain structure and function and often lead to persistent cognitive, emotional, and behavioral impairments, as well as long-term functional disability (Maas et al., 2008; Corrigan and Hammond, 2013; Ciurli et al., 2011).

In addition to neurological consequences, ABI is frequently associated with psychiatric complications. A substantial proportion of patients develop emotional disorders, including anxiety, depression, and post-traumatic stress symptoms (PTSS), which may negatively affect rehabilitation outcomes and quality of life (Meroni et al., 2013; Schwarzbold et al., 2008; Edmondson et al., 2013; Stein et al., 2018; Rutovic et al., 2019). Within this framework, Post-Traumatic Stress Disorder (PTSD) has emerged as a relevant clinical condition following ABI. PTSD may arise after exposure to a traumatic event and is characterized by intrusive symptoms, avoidance, negative alterations in cognition and mood, and persistent alterations in arousal and reactivity, leading to clinically significant distress and functional impairment (American Psychiatric Association, 2013).

However, the relationship between ABI and PTSD is characterized by a well-known clinical paradox. On the one hand, PTSD is typically associated with the formation and persistence of traumatic memories, which underlie re-experiencing symptoms. On the other hand, severe ABI is often accompanied by a period of loss of consciousness followed by post-traumatic amnesia (PTA) or post-lesional amnesia—the latter referring to amnesia of non-traumatic origin (e.g., vascular, anoxic, or tumor-related) —both representing severe neurobehavioral conditions during which the individual is unable to form continuous memories (Russell, 1935). For the sake of simplicity, the term PTA will be used hereafter to refer to both conditions.

Based on this assumption, it has traditionally been hypothesized that PTA may reduce the likelihood of developing PTSD, particularly symptoms related to re-experiencing (Sbordone, 1992; Price, 1994; Mayou et al., 1993; Sbordone and Liter, 1995; Warden et al., 1997; Glaesser et al., 2004). In line with this view, shorter PTA duration has been identified as a potential risk factor for PTSS following brain injury (Al-Ozairi et al., 2015).

Nevertheless, growing evidence challenges the “amnesia-protection” hypothesis. Several studies have shown that PTSS may emerge even in individuals with limited or absent explicit memory of the traumatic event (McMillan, 1991; Bryant and Harvey, 1995, 1999; Hickling et al., 1998; Layton and Wardi-Zonna, 1995; Bryant, 1996; Mayou et al., 2000; Feinstein et al., 2002; Williams et al., 2002; Harvey et al., 2003; McMillan et al., 2003; Bryant et al., 2009; Qureshi et al., 2019; see also the recent review by Villalobos and Bivona, 2021). Furthermore, the neuroanatomical and phenomenological overlap between ABI and PTSD may complicate differential diagnosis, making it challenging to disentangle trauma-related symptoms from the direct consequences of brain injury (Bryant, 2011; Motzkin and Koenigs, 2015). Accordingly, the relationship between PTA and PTSS following ABI may be more complex than traditionally assumed.

For this reason, the role of PTA in shaping the clinical expression of PTSS following ABI remains incompletely understood.

In addition to trauma-related memory processes, other clinical factors may contribute to the variability of PTSS following ABI. For instance, functional disability, reflecting the overall impact of brain injury on cognitive, behavioral, and everyday functioning, represents a key indicator of long-term outcome following ABI (Maas et al., 2008; Ponsford et al., 2008). Greater levels of disability have been associated with increased emotional distress and poorer psychosocial adjustment (Ponsford et al., 2008, 2014; Corrigan and Hammond, 2013; Ownsworth and Clare, 2006). At the same time, impaired self-awareness (ISA), is common after moderate-to-severe TBI and may affect the perception and reporting of emotional experiences (Prigatano, 1996; Sherer et al., 2003; Ownsworth and Clare, 2006). Thus, the subjective experience of disability—particularly when self-awareness is preserved—may influence the emotional processing of the traumatic event and contribute to PTSS (Bandiera et al., 2025). However, the role of these factors in PTSS following ABI remains unclear.

The present study aimed to investigate the relationship between PTA and the clinical expression of PTSS in ABI patients. Specifically, we examined whether the presence of PTA influences the manifestation of specific PTSS, rather than simply protecting against the development of PTSD. In addition, to better understand the mechanisms underlying PTSS expression following ABI, we explored the association between PTSS and relevant clinical variables, including functional disability and self-awareness.

## 2 Materials and methods

### 2.1 Study design

This study employed a cross-sectional observational design.

### 2.2 Participants

#### 2.2.1 Patients

Sixty patients with ABI of different etiologies were consecutively admitted to the Post-Coma Unit of the Santa Lucia Foundation (Rome, Italy) between June 2022 and October 2024. After enrolment, four patients were excluded: two patients due to lack of availability of an informal caregiver (hereafter referred to as “caregiver”), and two patients due to fatigue.

Therefore, 56 patients with ABI [TBI (*n* = 14), hemorrhage (*n* = 17), and ischemia (*n* = 25)] were finally included in the study according to the following inclusion and exclusion criteria:

*Inclusion criteria*:

Age ≥ 18 years; diagnosis of moderate or severe ABI defined by acute Glasgow Coma Scale (GCS; Teasdale and Jennett, 1974) score (namely, GCS 9–12 = moderate; ≤ 8 = severe); admission to intensive neurorehabilitation; and provision of written informed consent.

*Exclusion criteria*:

Previous or current substance abuse; previous or current psychiatric and/or neurological disease; current severe aphasia preventing psychological assessment; and distance from onset superior to 500 days.

Based on injury severity and PTA status, patients were divided into two groups: (1) patients with *severe* ABI who had emerged from PTA (“PTA group”) at the time of assessment; and (2) patients with *moderate* ABI (“No-PTA group”), namely, patients with no history of PTA.

All patients underwent routine clinical neuropsychological evaluation as part of standard clinical care.

The PTA group included 31 patients (20 males and 11 females), with a mean age of 38 years (SD = 15.6), a mean educational level of 13 years (SD = 2.7), a mean time since injury of 184 days (SD = 124.4), and a mean PTA duration of 44 days (SD = 35.5).

The No-PTA group consisted of 25 patients (17 males and 8 females), with a mean age of 55 years (SD = 9.7), a mean educational level of 14 years (SD = 3.5), and a mean time since injury of 79 days (SD = 65.9).

Forty-five out of 56 participants were inpatients, while 11 were undergoing neurorehabilitation treatment in a day hospital (DH) regimen.

#### 2.2.2 Caregivers

To evaluate patients' level of self-awareness, based on the discrepancy between patients' self-reports and caregivers' reports on the Patient Competency Rating Scale—Neurorehabilitation Form (PCRS-NR) (Borgaro and Prigatano, 2003), 56 first-degree relatives (aged ≥ 18 years) were enrolled.

Seventeen caregivers (30.4%) were parents of the patients, 23 (41.1%) were partners, 8 (14.3%) were adult children, and 8 (14.3%) were siblings. All relatives acted as primary caregivers (i.e., they continuously assisted the patient either in the hospital or at home during day-hospital treatment) and therefore had extensive knowledge of the patient's functioning.

All participants signed a written informed consent form prior to participation.

The study was approved by the Ethics Committee of Santa Lucia Foundation (Project identification code: CE/Prog.880; approval date: 28 November 2022) and was conducted in accordance with Good Clinical Practice (GCP) guidelines.

### 2.3 Research tools

#### 2.3.1 Procedure

After enrolment, all participants were fully informed about the aims and procedures of the study, and written informed consent was obtained.

Each patient was assessed by a psychologist in one or two sessions, depending on their level of fatigue, in order to complete all the questionnaires related to the variables of interest (see below).

Clinical data for patients were obtained from their medical records. Specifically, the following information was extracted: (1) Glasgow Coma Scale (GCS) score; (2) Level of Cognitive Functioning (LCF) score (Hagen et al., 1972); (3) Disability Rating Scale (DRS) score (Rappaport et al., 1982; Scranton et al., 1970); and (4) PTA duration, which was retrospectively estimated with caregiver support, a procedure commonly used when prospective PTA assessment is unavailable (McMillan et al., 1996).

To assess self-awareness, a different psychologist administered to one caregiver of each patient the relative questionnaire in one session in a quiet room.

#### 2.3.2 Patients' functional assessment

Patients' brain injury severity in the acute phase was determined using the GCS, which categorizes patients into three levels according to the following score ranges: 3–8 = severe; 9–12 = moderate; and 13–15 = mild.

Functional disability at the time of the interview was assessed using two widely employed scales in ABI populations: the LCF scale and the DRS.

The LCF evaluates patients' cognitive–behavioral functioning and ranges from 1 (No Response) to 8 (Purposeful/Appropriate Response). The DRS evaluates functional disability across three progressive domains—impairment, disability, and handicap—with total scores ranging from 0 (No disability) to 29 (Extreme Vegetative State). Time since injury (in days) was considered as time interval from ABI onset to the time of enrolment; coma duration was evaluated as time to follow commands (TFC).

#### 2.3.3 Post-traumatic stress disorder and post-traumatic stress symptoms

To assess the traumatic impact of the neurological event according to DSM-5 criteria, the Structured Clinical Interview for DSM-5—Clinician Version (SCID-5-CV) (American Psychiatric Association, 2013) was administered.

The SCID-5-CV is a semi-structured diagnostic interview used to assess DSM-5 psychiatric disorders. It is administered by trained clinicians and combines standardized questions with clinical judgment to ensure reliable and comprehensive diagnostic evaluation.

In the present study, Module G was administered to determine the presence of PTSD, including exposure to a traumatic event (Criterion A), intrusion symptoms (Criterion B), avoidance (Criterion C), negative alterations in cognition and mood (Criterion D), and alterations in arousal and reactivity (Criterion E), together with required duration (Criterion F), functional impairment (Criterion G), and exclusion criteria (Criterion H).

Beyond categorical diagnosis, dimensional symptom severity (namely, PTSS) was also examined. In line with dimensional approaches to PTSD assessment (Shankman et al., 2018) mean severity scores for Criteria B through E were calculated by summing item scores within each criterion and dividing by the number of endorsed items.

#### 2.3.4 Self-awareness assessment

To evaluate ISA in both inpatients and day-hospital (DH) patients, a shorter version of the PCRS, specifically designed for inpatient neurorehabilitation settings (Borgaro and Prigatano, 2003), namely the PCRS-NR, was administered.

The Patient Competency Rating Scale (PCRS) was used to assess self-awareness (Prigatano, 1986; Italian validation: Ciurli et al., 2010), given its well-established psychometric properties and feasibility in ABI populations (Smeets et al., 2012; Hellebrekers et al., 2017). It is a 30-item self-report questionnaire requiring both patients and their relatives to independently rate the patient's perceived level of competency across several behavioral, cognitive, and emotional situations using a 5-point Likert scale (ranging from 1, “Can't do,” to 5, “Can do with ease”). Total PCRS scores range from 30 to 150, with higher scores indicating higher levels of perceived competency.

The level of patients' metacognitive self-awareness was calculated using the PCRS discrepancy score (PCRS-NR-DS), obtained by subtracting the patient's total PCRS score from the relative's total PCRS score (Bivona et al., 2019; Prigatano, 1986).

The PCRS-NR excludes, instead, items referring to patients' competencies in activities of daily living outside the hospital environment. Accordingly, it assesses self-awareness across three domains: cognitive (three items), interpersonal (five items), and emotional (five items). Total PCRS-NR scores range from 13 to 65.

In line with previous studies (Bivona et al., 2022; Prigatano et al., 1998), a PCRS-NR discrepancy score (PCRS-NR-DS) cut-off of 5 points was adopted (scores ≥ 5 indicating low self-awareness) to distinguish between patients with high and low levels of self-awareness.

#### 2.3.5 Anxiety assessment

##### 2.3.5.1 Anxiety

The State–Trait Anxiety Inventory (STAI) (Spielberger et al., 1983) was used to assess self-reported anxiety levels. This instrument distinguishes between state anxiety (STAI-X1), which reflects transient anxiety experienced at the time of assessment, and trait anxiety (STAI-X2), which represents a relatively stable predisposition to experience anxiety across situations.

Each subscale includes 20 items, yielding total scores ranging from 20 (minimal anxiety) to 80 (maximal anxiety). Scores ≥ 40 on either subscale are generally considered indicative of clinically relevant anxiety.

## 3 Statistical analysis

Statistical analyses were performed in Jamovi (Version 2.6.25.0). Assumption checks are available in the Supplementary material. Group differences (PTA vs. No-PTA) were tested using two-tailed Welch's independent-samples *t*-test when normality was acceptable but the homogeneity of variances assumption was violated, Mann-Whitney U tests when the normality assumption was violated, and Students' independent samples *t*-tests when both assumptions were met. For comparisons across the four SCID-5 symptom subdimensions, the family-wise error rate was controlled using a Bonferroni-corrected threshold (α = 0.05/4 = 0.0125).

Within each group, bivariate associations among SCID-5-CV subdimensions, DRS, PCRS-NR-DS, Time since injury as well as TFC and PTA (exclusively for the PTA group) were examined using Pearson's correlations (two-tailed). Correlation analyses were considered exploratory and uncorrected *p*-values were reported.

To examine whether the associations between PTSS and arousal symptoms differed between groups, a hierarchical multiple regression was fitted with SCID-5-CV arousal as the dependent variable. Model 1 included Group and time since injury; Model 2 added the main effects of PCRS-NR-DS and DRS; Model 3 added the Group × PCRS-NR-DS and Group × DRS interaction terms; Model 4 additionally included age and sex as covariates. Continuous predictors were standardized prior to computing interactions. Models were compared using *R*^2^/Δ*R*^2^ and AIC; incremental variance explained was evaluated via the change in model fit (Δ*R*^2^ test). Regression assumptions (normality of residuals, homoscedasticity, autocorrelation, and multicollinearity) were met and are reported in the Supplementary material. To follow up significant interactions in Model 3, we used estimated marginal means (EMM) to estimate group differences at representative values of the continuous predictors (mean and ±1 SD) and to test simple slopes within each group.

## 4 Results

### 4.1 PTSD diagnosis and related symptoms

The two groups (PTA vs. No-PTA) differed significantly in terms of time since injury, with the PTA patients showing a longer time since the ABI [Welch's *t*_(47.4)_ = −4.05, *p* < 0.001], as well as in terms of age, with PTA patients being significantly younger than No-PTA patients [Welch's *t*_(51)_ = 5, *p* < 0.001]. However, the groups did not significantly differ in overall symptom severity as measured by the DRS (Mann–Whitney *U* = 280, *p* = 0.075).

According to the SCID-5-CV interview, only one patient in the PTA group met criteria for a current diagnosis of PTSD, while one patient had previously met criteria for PTSD related to the traumatic event. No other PTSD diagnoses were identified.

**Figure 1:**
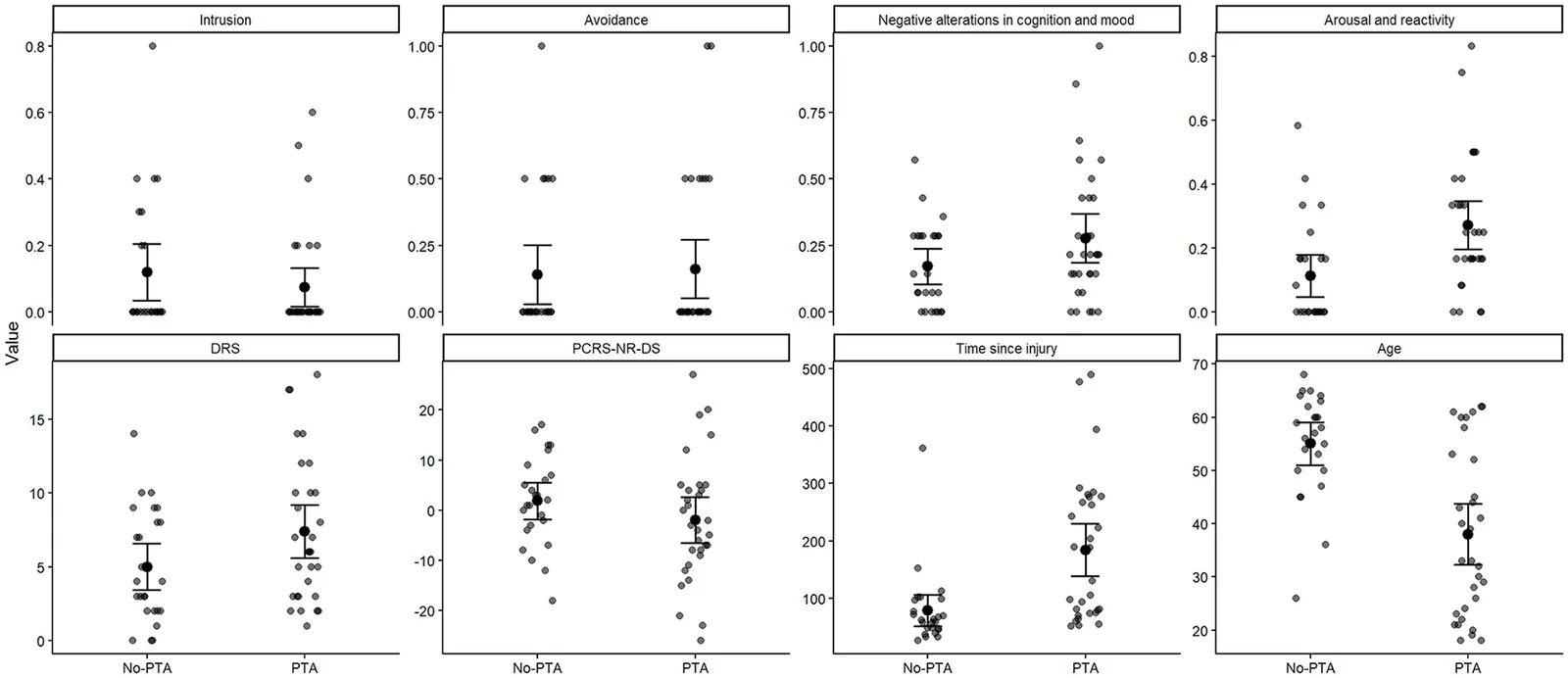
Group differences between PTA and No-PTA patients. Dots represent individual participants; larger points and error bars represent group means and 95% confidence intervals. PTSS cluster scores are reported as item-averaged scores to allow comparability across clusters with different numbers of items.

When comparing the two groups on the PTSS, a significant difference emerged in the arousal and reactivity domain (criterion E) (Mann–Whitney *U* = 198, *p* < 0.001). This effect remained significant after Bonferroni correction for multiple comparisons (Bonferroni-corrected α = 0.0125). Specifically, patients in the PTA group reported significantly higher levels of arousal/reactivity symptoms (see Table 1). A graphical representation of the group comparisons is provided in Figure 1, which displays the distribution of individual observations together with group means and 95% confidence intervals for each variable.

**Table 1:** Comparisons between the PTA and No-PTA groups on PTSS, overall symptom severity (DRS) and ISA (PCRS-NR-DS).

Variable	PTA M(SD)	No-PTA M(SD)	Test	Statistic	*P*
Intrusion	0.07 (0.16)	1.20 (2.06)	Mann–Whitney *U*	347	0.391
Avoidance	0.16 (0.3)	0.14 (0.27)	Mann–Whitney *U*	379	0.853
Negative alterations in cognition and mood	0.28 (0.25)	0.17 (0.16)	Mann–Whitney *U*	304	0.166
Arousal and reactivity	0.27 (0.21)	0.113 (0.16)	Mann–Whitney *U*	198	0.001
DRS	7.39 (4.9)	5 (3.8)	Mann–Whitney *U*	280	0.075
PCRS-NR-DS	−1.903 (−3)	1.88 (8.85)	Students' *t*	df = 54, *t* = 1.29	0.204
Time since injury (days)	183.77 (124.44)	78.6 (65.94)	Welch's *t*	df = 47.4, *t* = −4.05	< 0.001
Age (years)	38 (15.64)	55 (9.73)	Welch's *t*	df = 51, *t* = 5	< 0.001

PTA, post-traumatic amnesia; No-PTA, patients without PTA; PTSS, post-traumatic stress symptoms; DRS, disability rating scale; PCRS-NR-DS, patient competency rating scale-neurorehabilitation form–discrepancy scores (measure of ISA). SCID-5-CV symptom-cluster scores are reported as item-averaged scores, computed by summing the items within each symptom cluster and dividing by the number of items in that cluster, to allow comparability across clusters with different item numbers. DRS, PCRS-NR-DS, age, and time since injury are reported on their original scales.

As for anxiety, given the violation of normality assumptions, group differences in STAI scores were examined using the Mann–Whitney *U* test. No significant differences emerged between PTA and non-PTA patients for either STAI X-1 (*U* = 363, *p* = 0.692) or STAI X-2 (*U* = 383, *p* = 0.947), indicating similar levels (slightly below the clinical cut-offs) of state and trait anxiety across the two groups (see Supplementary material for details).

### 4.2 Correlations

Separate correlation analyses were performed within the PTA and No-PTA groups (see Tables 2, 3, respectively).

**Table 2:** Correlation matrix for the PTA group (df = 29).

		Intrusion	Avoidance	Negative alterations in cognition and mood	Arousal and reactivity	DRS	PCRS-NR-DS	Time since injury	TFC	PTA
Intrusion	Pearson's *r*	—								
	*p*-value	—								
Avoidance	Pearson's *r*	0.230	—							
	*p*-value	0.213	—							
Negative alterations in cognition and mood	Pearson's *r*	0.280	0.417^*^	—						
	*p*-value	0.127	0.020	—						
Arousal and reactivity	Pearson's *r*	0.407^*^	−0.036	0.464^**^	—					
	*p*-value	0.023	0.850	0.009	—					
DRS	Pearson's *r*	0.022	0.206	−0.286	−0.242	—				
	*p*-value	0.907	0.267	0.119	0.190	—				
PCRS-NR-DS	Pearson's *r*	−0.210	0.009	−0.347	−0.413^*^	0.007	—			
	*p*-value	0.256	0.961	0.056	0.021	0.970	—			
Time since injury	Pearson's *r*	0.111	−0.049	0.198	0.560^**^	−0.226	−0.124	—		
	*p*-value	0.553	0.795	0.285	0.001	0.223	0.508	—		
TFC	Pearson's *r*	0.274	−0.037	0.076	0.180	−0.050	−0.267	0.338	—	
	*p*-value	0.136	0.843	0.684	0.332	0.791	0.146	0.063	—	
PTA	Pearson's *r*	0.217	−0.006	0.144	0.177	−0.060	−0.136	0.444^*^	0.594^***^	—
	*p*-value	0.240	0.973	0.439	0.341	0.749	0.466	0.012	< 0.001	—

^*^p < 0.05, ^**^p < 0.01, ^***^p < 0.001. PTA, post-traumatic amnesia; DRS, disability rating scale; PCRS-NR-DS, patient competency rating scale-neurorehabilitation form– discrepancy scores (measure of ISA); TFC, time to follow commands.

**Table 3:** Correlation matrix for the No-PTA group (df = 23).

		Intrusion	Avoidance	Negative alterations in cognition and mood	Arousal and reactivity	DRS	PCRS-NR-DS	Time since injury
Intrusion	Pearson's *r*	—						
	*p*-value	—						
Avoidance	Pearson's *r*	0.284	—					
	*p*-value	0.169	—					
Negative alterations in cognition and mood	Pearson's *r*	0.323	0.348	—				
	*p*-value	0.116	0.088	—				
Arousal and reactivity	Pearson's *r*	0.148	0.138	0.711^***^	—			
	*p*-value	0.479	0.509	< 0.001	—			
DRS	Pearson's *r*	0.069	0.403^*^	0.590^**^	0.575^**^	—		
	*p*-value	0.744	0.046	0.002	0.003	—		
PCRS-NR-DS	Pearson's *r*	−0.035	−0.106	−0.210	−0.078	−0.025	—	
	*p*-value	0.867	0.615	0.313	0.712	0.907	—	
Time since injury	Pearson's *r*	0.100	−0.092	0.441^*^	0.427^*^	0.334	0.120	—
	*p*-value	0.636	0.660	0.027	0.033	0.103	0.569	—

^*^p < 0.05, ^**^p < 0.01, ^***^p < 0.001. PTA, post-traumatic amnesia; DRS, disability rating scale; PCRS-NR-DS, patient competency rating scale-neurorehabilitation form– discrepancy scores (measure of ISA).

Time since injury showed a significant positive correlation with arousal symptoms both in No-PTA (*r* = 0.437, *p* = 0.033) and in PTA (*r* = 0.56, *p* = 0.001). In contrast, PTA duration did not significantly correlate with any PTSS.

Within the PTA group, ISA (namely, higher PCRS-NR-DS) were not significantly correlated with PTA duration, that is higher PCRS-NR-DS were not associated with longer PTA duration. However, greater PCRS-NR-DS were significantly negatively correlated with arousal symptoms (*r* = −0.413, *p* = 0.021), whereas no significant linear relationships emerged with the other PTSS (see Table 2). DRS scores did not show significant correlations with any of the PTSS.

Conversely, in the No-PTA group (Table 3), ISA was not significantly associated with any of the considered variables. DRS scores were positively correlated with avoidance (*r* = 0.403, *p* = 0.046), negative mood and cognition (*r* = 0.590, *p* = 0.002), and arousal symptoms (*r* = 0.575, *p* = 0.003), but not with intrusion symptoms.

Inspection of the correlation matrices suggests a differential pattern between groups. In the PTA group, more severe ISA (PCRS-NR-DS) was associated with lower levels of arousal symptoms, whereas DRS scores were unrelated to arousal. Conversely, in the No-PTA group, higher DRS scores were associated with greater PTSS in terms of avoidance, negative mood and cognition, and arousal, while ISA showed no significant associations. This pattern suggests that the relationship between arousal symptoms and clinical variables may differ as a function of the presence of PTA.

### 4.3 Hierarchical multiple regression

To further investigate whether the relationship between arousal symptoms and clinical variables differed according to PTA presence/absence, a hierarchical multiple regression analysis was performed with arousal symptoms as the dependent variable.

The base model included patients' group and time since injury as predictors. The model including the interaction terms model accounted for 52.1% of the variance in arousal symptoms (*R*^2^ = 0.52). Indeed, the inclusion of interaction terms (Group × PCRS-NR-DS and Group × DRS) significantly improved model fit (Δ*R*^2^ = 0.07, *p* = 0.035) compared with the model including only main effects. Adding age and sex in the subsequent covariate-adjusted model did not significantly improve model fit (Δ*R*^2^ = 0.02, *p* = 0.34) and neither age nor sex emerged as a significant predictor. This suggests that the observed associations were not accounted for by individual differences in age or sex.

Time since injury emerged as a significant positive predictor of hyper-arousal across groups (*p* < 0.001). Although DRS scores did not show a significant main effect (*p* = 0.328), a significant Group × DRS interaction was observed (b = 1.38, SE = 0.55, *p* = 0.014), indicating that the association between overall disability and arousal differed between groups. Specifically, higher DRS scores (indicating higher level of disability) were associated with increased arousal symptoms in No-PTA patients, whereas no significant association emerged in the PTA group.

Conversely, PCRS-NR-DS showed a significant main effect on arousal symptoms (*b* = −0.78, SE = 0.29, *p* = 0.01), but the Group × PCRS-NR-DS interaction was not significant (*p* = 0.334; see Table 4).

**Table 4:** Multiple regression model.

Predictor	Estimate	SE	95% confidence interval		*t*	*p*
			Lower	Upper		
Intercept^a^	2.767	0.353	2.057	3.476	7.837	< 0.001
Patients						
No-PTA – PTA	−0.498	0.568	−1.639	0.644	−0.877	0.385
Time since injury	1.081	0.281	0.516	1.646	3.845	< 0.001
DRS	−0.306	0.309	−0.928	0.316	−0.988	0.328
PCRS-NR-DS	−0.779	0.291	−1.364	−0.194	−2.678	0.010
DRS × patients						
DRS × (No-PTA – PTA)	1.384	0.545	0.287	2.480	2.537	0.014
PCRS-NR-DS × patients						
PCRS-NR-DS × (No-PTA – PTA)	0.527	0.540	−0.557	1.611	0.976	0.334

PTA, post-traumatic amnesia; DRS, disability rating scale; PCRS-NR-DS, patient competency rating scale-neurorehabilitation form– discrepancy scores. ^a^Represents reference level. Continuous predictors were standardized.

**Figure 2:**
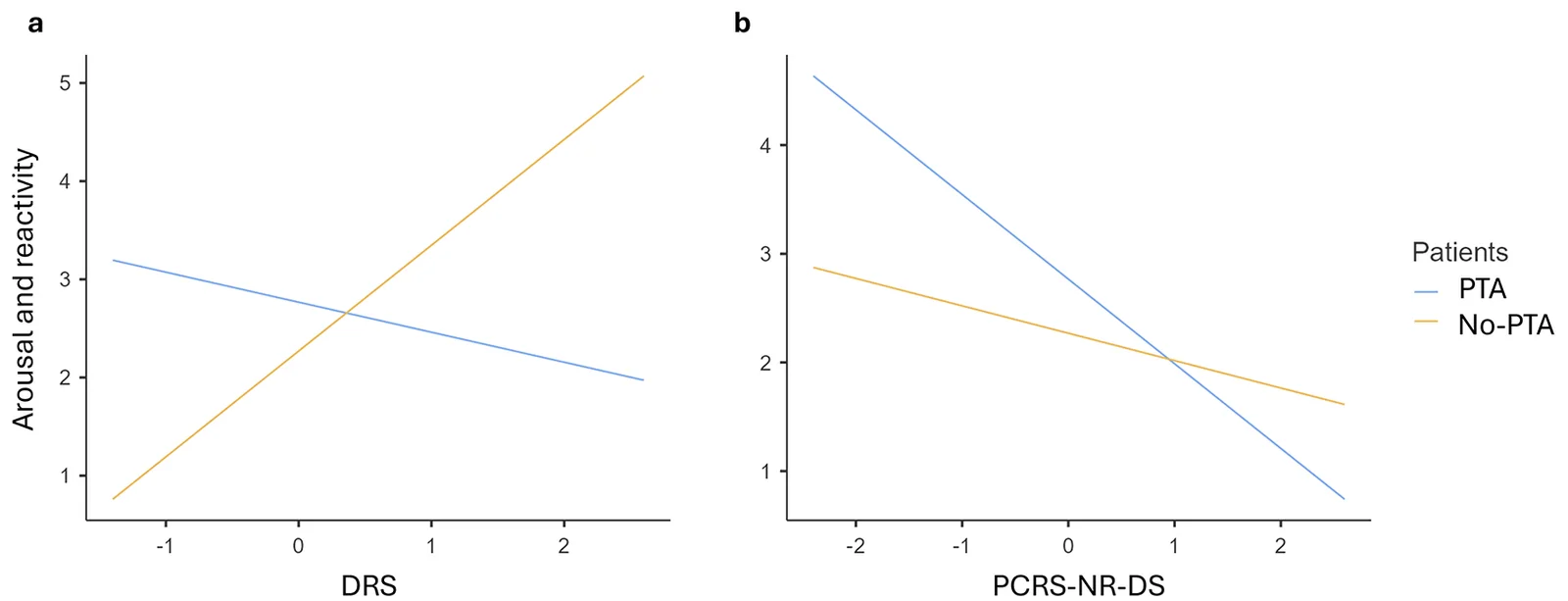
Estimated marginal means of arousal symptoms by patient group. **(a)** Interaction between overall severity scores (DRS) and patient group. **(b)** Interaction between ISA scores (PCRS-NR-DS) and patient group.

EMMs were examined to facilitate interpretation of the interaction terms. As shown in Figure 2a, illustrating the significant Groups × DRS interaction, higher functional disability was associated with increased arousal symptoms in No-PTA patients, whereas arousal symptoms slightly decreased with increasing functional disability in PTA patients. Conversely, the non-significant Groups × PCRS-NR-DS interaction indicated that arousal symptoms decreased with increasing ISA scores in both groups (Figure 2b).

## 5 Discussion

### 5.1 PTA and the expression of post-traumatic stress symptoms

The present study investigated whether PTA influences the clinical expression of PTSS following ABI. Contrary to the traditional “amnesia-protection” hypothesis (Sbordone, 1992; Mayou et al., 1993; Sbordone and Liter, 1995; Warden et al., 1997; Glaesser et al., 2004), our findings suggest that PTA does not necessarily reduce trauma-related symptomatology following ABI.

Importantly, although categorical PTSD diagnoses were rare in both groups, patients with PTA reported significantly higher arousal/reactivity symptoms compared to patients without PTA, whereas no significant differences emerged for intrusion, avoidance, or negative cognition/mood symptoms. These findings suggest that PTA may be associated with a selective alteration of PTSS expression rather than with a generalized reduction of trauma-related symptoms.

This interpretation is further supported by the absence of significant differences in state and trait anxiety between groups. Specifically, STAI scores were comparable and remained below clinical cut-offs in both groups, suggesting that the increased arousal symptoms observed in PTA patients were unlikely to merely reflect generalized anxiety, but rather a more specific dimension of post-traumatic symptomatology following ABI.

### 5.2 Trauma memory and physiological arousal following ABI

One possible interpretation of the present findings concerns the relationship between trauma memory and physiological arousal following ABI. Previous studies suggested that the encoding and consolidation of the traumatic event play an important role in the development of PTSS following brain injury (Sbordone, 1992; Mayou et al., 1993; Sbordone and Liter, 1995; Warden et al., 1997; Glaesser et al., 2004). In particular, patients who retain autobiographical memory of the traumatic event may show a greater risk of developing PTSS (Gil et al., 2005).

However, growing evidence indicates that trauma-related symptoms may also emerge in the absence of complete explicit recollection of the traumatic event (Bryant and Harvey, 1995, 1999; Feinstein et al., 2002; Harvey et al., 2003; McMillan et al., 2003; Bryant et al., 2009). Within this framework, PTA may impair the autobiographical encoding and retrieval of the traumatic experience while leaving relatively preserved the neurobiological systems involved in emotional arousal and threat responsivity (Bryant, 2001, 2011).

Accordingly, the increased arousal symptoms we observed in PTA patients may reflect a partial dissociation between impaired declarative memory processes and relatively preserved autonomic and emotional responses to trauma. This interpretation is consistent with theoretical models suggesting that conditioned physiological responses to traumatic experiences may develop even when explicit autobiographical memory is fragmented or inaccessible (Bryant, 2001, 2011; Villalobos and Bivona, 2021).

### 5.3 Functional disability, self-awareness, and PTSS

The present findings also suggest that functional disability and self-awareness may differentially contribute to the expression of PTSS following ABI. In patients without PTA, greater functional disability was associated with higher levels of arousal symptoms, as well as with avoidance and negative alterations in cognition and mood. This pattern is consistent with previous evidence linking greater disability after ABI with increased emotional distress and poorer psychosocial adjustment (Ponsford et al., 2008, 2014; Corrigan and Hammond, 2013; Ownsworth and Clare, 2006).

One possible interpretation is that, when the traumatic event and its consequences remain cognitively accessible, functional disability may become more directly integrated into the individual's emotional appraisal of the injury, thereby contributing to trauma-related distress. Conversely, this association was not observed in patients with PTA, suggesting that disruption of autobiographical memory processes may weaken the relationship between functional impairment and emotional responses to trauma.

Importantly, although levels of ISA did not differ between groups, it emerged as a significant negative predictor of arousal symptoms across groups. This finding suggests that reduced self-awareness may influence the subjective recognition and reporting of emotional and physiological symptoms following ABI. Previous studies have shown that ISA frequently affects the monitoring and appraisal of cognitive, behavioral, and emotional difficulties after brain injury (Prigatano, 1996; Sherer et al., 2003; Ownsworth and Clare, 2006; Ciurli et al., 2010; Bivona et al., 2019, 2022). Accordingly, lower reported levels of arousal in patients with greater ISA may not necessarily reflect reduced physiological activation *per se*, but rather diminished awareness and reporting of internal emotional states.

### 5.4 Clinical implications

The present findings may also have relevant clinical implications for the assessment of post-traumatic stress symptoms following ABI. In the current study, PTSS were evaluated using a structured clinical interview (SCID-5-CV) rather than self-report questionnaires. This methodological choice was particularly important given that impaired self-awareness is common after moderate-to-severe ABI and may affect the recognition and reporting of emotional symptoms (Prigatano, 1996; Sherer et al., 2003; Ownsworth and Clare, 2006). In addition, previous studies highlighted the diagnostic complexity of distinguishing trauma-related symptoms from the neuropsychiatric consequences of brain injury itself (McMillan, 2001; Bryant, 2011; Alway et al., 2016).

The negative association observed between ISA and arousal symptoms further suggests that trauma-related distress may sometimes be under-recognized or under-reported in ABI populations. Accordingly, assessment of PTSS following ABI may benefit from multidimensional clinical approaches integrating structured interviews, neuropsychological assessment, caregiver information, and measures of self-awareness.

More broadly, the present findings highlight the importance of considering trauma-related symptoms even in patients with PTA and fragmented autobiographical memory of the traumatic event. Clinicians should therefore avoid assuming that impaired trauma recollection necessarily reflects reduced emotional impact of the injury experience.

### 5.5 Limitations and future directions

Several limitations should be considered when interpreting the present findings.

First, the relatively limited sample size may have reduced the statistical power to detect smaller effects across PTSS dimensions. In addition, PTSD diagnoses were rare in both groups, potentially contributing to floor effects and limiting the possibility of identifying broader differences in categorical PTSD outcomes. Future studies should consider recruiting larger samples, including individuals with more severe ABI, to address this limitation.

Second, although exploratory analyses were conducted to evaluate potential age and sex effects, the two groups differed in mean age, and this aspect should be considered when interpreting the findings. Future studies with larger and more demographically balanced samples are needed to further clarify the contribution of these variables.

Third, the cross-sectional design does not allow conclusions regarding the temporal evolution or causal mechanisms underlying the relationship between PTA and PTSS. Longitudinal studies could help clarify how trauma-related symptoms develop over time following ABI and whether different PTSS dimensions follow distinct trajectories.

Moreover, the inclusion of patients with heterogeneous ABI etiologies may have contributed additional clinical variability. Future research should examine these processes in more homogeneous clinical populations and further investigate the neuropsychological and neurobiological mechanisms linking autobiographical memory, emotional arousal, and trauma-related symptoms following ABI.

Finally, although PTSS were assessed through a structured clinical interview, the interpretation of trauma-related symptoms following ABI remains clinically complex, particularly in the presence of impaired self-awareness and cognitive dysfunction. Future studies integrating neuropsychological, psychophysiological (e.g., autonomic or neuroendocrine indices), and caregiver-based assessments may provide a more comprehensive understanding of post-traumatic symptom expression following brain injury.

However, despite these limitations, the present study provides novel insights into the relationship between PTA and trauma-related symptom expression following ABI.

To our knowledge, this is among the few studies to specifically investigate how PTA may differentially relate to distinct dimensions of PTSS rather than to PTSD diagnosis alone. In particular, the finding of increased arousal/reactivity symptoms in patients with PTA challenges the traditional “amnesia-protection” hypothesis and supports the possibility that trauma-related emotional and physiological responses may persist even in the absence of complete autobiographical recollection of the traumatic event.

Furthermore, by integrating measures of functional disability and self-awareness, the present study highlights the importance of considering metacognitive and neuropsychological factors in the assessment of PTSS following ABI. Overall, these findings may contribute to a more nuanced understanding of PTSS after ABI and encourage the development of multidimensional assessment approaches in both clinical and research settings.

## Data Availability

The raw data supporting the conclusions of this article will be made available by the authors, without undue reservation.
