# Supplementary material for: Post-traumatic amnesia is associated with increased arousal and reactivity symptoms following acquired brain injury
Source: Front Cognit. 2026 Jul 28;5:1880462. doi: 10.3389/fcogn.2026.1880462 (PMC13488039; doi:10.3389/fcogn.2026.1880462)
Supplement: Supplementary file 1 [file Supplementary_File_1.docx]

***Supplementary Material***

**Between groups comparisons’ assumptions checks**

S table 1. Normality checks.

|  | test | statistic | p |
| --- | --- | --- | --- |
| Intrusion | Shapiro-Wilk | 0.678 | <.001 |
|  | Anderson-Darling | 7.32 | <.001 |
| Avoidance | Shapiro-Wilk | 0.601 | <.001 |
|  | Anderson-Darling | 10.33 | <.001 |
| Negative alterations in cognition and mood | Shapiro-Wilk | 0.903 | <.001 |
|  | Anderson-Darling | 1.52 | <.001 |
| Arousal and reactivity | Shapiro-Wilk | 0.883 | <.001 |
|  | Anderson-Darling | 2.51 | <.001 |
| Time since injury (days) | Shapiro-Wilk | 0.887 | <.001 |
|  | Anderson-Darling | 1.53 | <.001 |
| DRS | Shapiro-Wilk | 0.931 | 0.003 |
|  | Anderson-Darling | 1.19 | 0.004 |
| PCRS-NR-DS | Shapiro-Wilk | 0.991 | 0.951 |
|  | Anderson-Darling | 0.179 | 0.913 |
| Age (y) | Shapiro-Wilk | 0.973 | 0.24 |
|  | Anderson-Darling | 0.360 | 0.438 |
|  | | | |

| S table 2. Homogeneity of variances checks. | | | | | | |
| --- | --- | --- | --- | --- | --- | --- |
|  | | | | | | |
|  | | | | | |  |
|  | Test | F | df | df2 | p |  |
| Intrusion | Levene's | 2.477 | 1 | 54 | 0.121 |  |
|  | Variance ratio | 1.679 | 24 | 30 | 0.178 |  |
| Avoidance | Levene's | 0.340 | 1 | 54 | 0.562 |  |
|  | Variance ratio | 0.817 | 24 | 30 | 0.616 |  |
| Negative alterations | Levene's | 2.207 | 1 | 54 | 0.143 |  |
|  | Variance ratio | 0.413 | 24 | 30 | 0.029 |  |
| Arousal and reactivity | Levene's | 1.084 | 1 | 54 | 0.302 |  |
|  | Variance ratio | 0.610 | 24 | 30 | 0.218 |  |
| Time since injury (days) | Levene's | 14.948 | 1 | 54 | <.001 |  |
|  | Variance ratio | 0.281 | 24 | 30 | 0.002 |  |
| DRS | Levene's | 1.372 | 1 | 54 | 0.247 |  |
|  | Variance ratio | 0.606 | 24 | 30 | 0.212 |  |
| PCRS-NR-DS | Levene's | 2.294 | 1 | 54 | 0.136 |  |
|  | Variance ratio | 0.511 | 24 | 30 | 0.096 |  |
| Age (y) | Levene's | 11.991 | 1 | 54 | 0.001 |  |
|  | Variance ratio | 0.387 | 24 | 30 | 0.02 |  |
|  | | | | | |  |

**Regression model assumptions checks**

S table 3. Normality assumptions

| Test | Statistic | p |
| --- | --- | --- |
| Shapiro-Wilk | 0.94 | 0.007 |
| Kolmogorov-Smirnov | 0.11 | 0.419 |
| Anderson-Darling | 0.78 | 0.040 |

S table 4. Homoscedasticity Tests

| Test | Statistic | p |
| --- | --- | --- |
| Breusch-Pagan | 5.03 | 0.755 |
| Goldfeld-Quandt | 0.52 | 0.919 |
| Harrison-McCabe | 0.60 | 0.887 |

S table 5. Durbin–Watson Test for Autocorrelation

| Autocorrelation | DW Statistic | p |
| --- | --- | --- |
| 0.11 | 1.76 | 0.286 |

S table 6. Collinearity Statistics

| Variable | VIF | Tolerance |
| --- | --- | --- |
| Patients | 1.96 | 0.51 |
| Time since injury | 1.58 | 0.63 |
| Sex | 1.28 | 0.78 |
| Age | 1.88 | 0.53 |
| DRS_s | 1.76 | 0.57 |
| PCRS-NR-DS_s | 1.90 | 0.53 |
| DRS_s ✻ Patients | 1.74 | 0.57 |
| PCRS-NR-DS_s ✻ Patients | 1.48 | 0.68 |

*Notes.* The s means that variables were standardized.
